# Supplementary material for: Molecular Mechanisms through Which Short-Term Cold Storage Improves the Nutritional Quality and Sensory Characteristics of Postharvest Sweet Potato Tuberous Roots: A Transcriptomic Study
Source: Foods. 2021 Sep 2;10(9):2079. doi: 10.3390/foods10092079 (PMC8469081; doi:10.3390/foods10092079)
Supplement: Supplementary file 1 [file foods-10-02079-s001.zip › Table S1-S7.pdf]

Table S1. Primers for Quantitative Real-time PCR

Gene	Forward primer (5'-3')	Reverse primer (5'-3')
α-Tubulin	CAACTACCAGCCACCAACTGT	CAAGATCCTCACGAGCTTCAC
itf11g07860	GGTGCGCCAATGGATCTCTA	CCAGCAATGAGGCAACAAGG
itf03g13310	TTCTGGTTGTCGCCGATGAA	GATGATCTCGTCTGGTCGGC
itf09g14800	CTTCGCGCAATTCTCTGAGC	GCTCTGGACGTGATTGGTGA
itf11g10280	GACGATGGCGAATCCTCTGT	AGTCGGTGCAGATCACCTTG
itf11g08270	CAGATTCTTGCCAGCCTCGT	CTTCGAGTGTCTTTGCACGC
itf09g04020	ACACTTTGTTGTGAGCGTGC	ACGATCACCAGGCAATAGCA
itf10g08090	TCCATCGGTGCCTCCTTCTA	GCGCTTGAGGCGTTGAATAG
itf07g12100	CCATCGCTCCATCTTCGAGT	GTTAGCAGCGTTGAAGCCAC

Table S2. Sample sequencing data evaluation statistics table

Samples	Read Number	Base Number	GC Content	%≥Q30
C0-1	31,991,228	9,513,061,176	47.09	94.31
C0-2	26,415,067	7,864,228,308	47.14	94.04
C0-3	23,197,468	6,905,479,554	47.11	94.18
C14-1	25,520,266	7,608,729,444	47.06	94.22
C14-2	25,863,789	7,694,022,216	47.21	94.40
C14-3	21,576,710	6,437,240,780	46.59	94.05
CS14-1	27,230,923	8,104,053,402	46.22	94.42
CS14-2	30,446,586	9,058,842,306	46.28	94.20
CS14-3	27,230,923	8,104,053,402	46.22	94.42

Table S3. Statistical table of comparison results between the second-generation sequencing data and the reference genome

Sample	Total Reads	Mapped reads	Uniquely mapped reads	Multiple mapped reads
C0-1	63,982,456	78.15%	75.00%	3.16%
C0-2	52,830,134	77.64%	74.64%	3.00%
C0-3	46,394,936	78.40%	75.98%	2.42%
C14-1	51,040,532	80.06%	77.39%	2.67%
C14-2	51,727,578	79.26%	75.83%	3.43%
C14-3	43,153,420	79.97%	77.40%	2.57%
CS14-1	54,461,846	82.57%	79.49%	3.08%
CS14-2	60,893,172	82.53%	79.31%	3.23%
CS14-3	55,639,714	82.17%	78.77%	3.40%

Table S4. Annotation statistics table of single gene sequence

Bionformation database	Annotated unigene number	300<=length <1000	length>=1000	Percentage (%)
COG	11147	3646	7373	35.78
GO	16472	6480	9521	52.88
KEGG	10975	4518	6131	35.23
KOG	17362	6364	10630	55.73
Pfam	26908	10515	15802	86.38
Swissprot	22909	8812	13564	73.54
eggNOG	29640	12125	16681	95.15
NR	31000	12968	17091	99.52
All_Annotated	31151	13078	17108	100

Table S5. Statistics of the number of annotations for differentially expressed genes

DEG set	Total	COG	eggNOG	NR	Pfam	Swiss-Prot	GO	KEGG
C0 vs C14	1,068	427	976	1,016	928	811	632	380
C0 vs CS14	9,936	3,981	9,261	9,509	8,370	7,106	5,551	3,697

Table S6. Comparison table of GO function annotations of differentially expressed genes

Go term	Go classify	C0 vs C14	C0 vs CS14	Ratio of DEGs
cellular component	cell	200	2436	12.18
	membrane	169	1352	8.00
	macromolecular complex	42	668	15.90
	organelle	149	1826	12.26
	organelle part	75	872	11.63
	membrane part	86	687	7.99
	cell part	201	2448	12.18
molecular function	catalytic activity	376	2982	7.93
	binding	301	2597	8.63
biological process	metabolic process	449	3764	8.38
	cellular process	324	3105	9.58
	single-organism process	338	2694	7.97
	response to stimulus	138	1153	8.36
	localization	111	883	7.95
	biological regulation	111	1017	9.16
	cellular component organization or biogenesis	55	580	10.55

Table S7. Function annotation comparison table of differentially expressed gene KEGG

KEGG function annotation	Number	C0 vs C14	C0 vs CS14	Differences
Plant hormone signal transduction	ko04075	18	100	82
Protein processing in endoplasmic reticulum	ko04141	4	108	104
Ubiquitin mediated proteolysis	ko04120	4	55	51
Ribosome	ko03010	3	225	222
Phenylpropanoid biosynthesis	ko00940	27	82	55
Amino sugar and nucleotide sugar metabolism	ko00520	7	69	62
Glycolysis / Gluconeogenesis	ko00010	10	76	66
Starch and sucrose metabolism	ko00500	11	107	96
Biosynthesis of amino acids	ko01230	16	111	95
Carbon metabolism	ko01200	11	131	120
Glutathione metabolism	ko00480	6	63	57
Purine metabolism	ko00230	6	82	76
Pyrimidine metabolism	ko00240	11	70	59
Plant-pathogen interaction	ko04626	13	84	71
Endocytosis	ko04144	0	58	58
Proteasome	ko03050	0	54	54
Spliceosome	ko03040	0	67	67
RNA transport	ko03013	0	68	68
Oxidative phosphorylation	ko00190	0	50	50
