# Supplementary figures and images for: Molecular Mechanisms through Which Short-Term Cold Storage Improves the Nutritional Quality and Sensory Characteristics of Postharvest Sweet Potato Tuberous Roots: A Transcriptomic Study
Source: Foods. 2021 Sep 2;10(9):2079. doi: 10.3390/foods10092079 (PMC8469081; doi:10.3390/foods10092079)

A

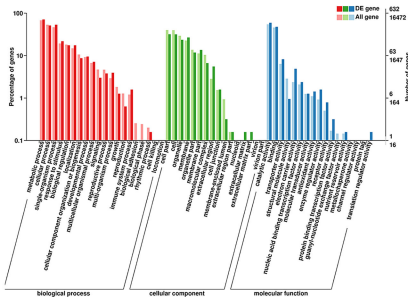

B

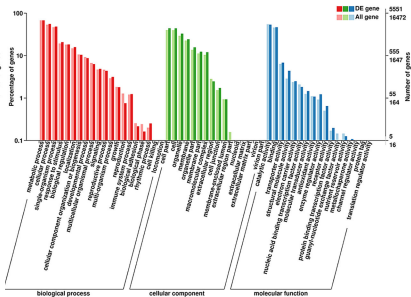

Supplement: Supplementary file 1 [file foods-10-02079-s001.zip › Figure S1.pdf]

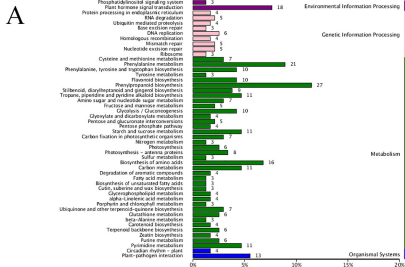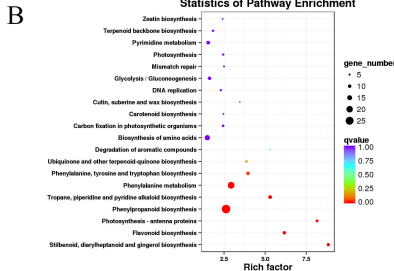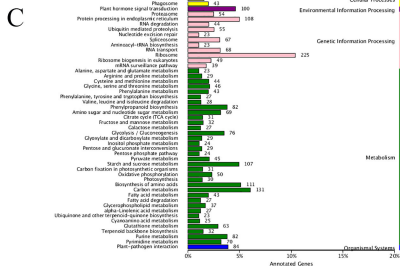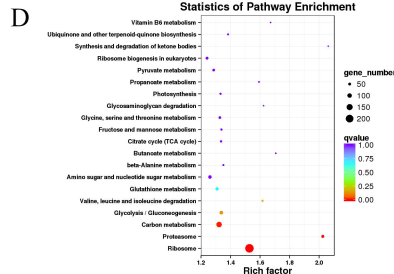

Supplement: Supplementary file 1 [file foods-10-02079-s001.zip › Figure S2.pdf]
